# Adult Chemical Pneumonitis Caused by Accidental Kerosene Aspiration: A Case Report

**DOI:** 10.7759/cureus.104383

**Published:** 2026-02-27

**Authors:** Kohei Watanabe, Jun Nakamura, Seiko Tanaka, Kazuhisa Nakashima

**Affiliations:** 1 Department of Respiratory Medicine, Shimane Prefectural Central Hospital, Izumo, JPN

**Keywords:** aspiration, chemical pneumonitis, kerosene, lung cavitation, petroleum distillate

## Abstract

Kerosene aspiration is a recognized cause of chemical pneumonitis, most commonly reported in children, whereas adult cases are rare. We present a case of severe chemical pneumonitis in an elderly patient following accidental kerosene ingestion in an agricultural setting. A 73-year-old male farmer undergoing maintenance dialysis accidentally ingested kerosene that had been stored in a plastic drink bottle during open-field burning. Although he immediately spat it out, he developed progressive dyspnea, fatigue, and vomiting. Chest radiography and computed tomography revealed extensive bilateral pulmonary infiltrates, predominantly in the lower lung fields. The patient was admitted to the intensive care unit for supportive management; mechanical ventilation was not required. Empirical antibiotic therapy was initiated due to concern for secondary infection. Serial imaging demonstrated evolution of the pulmonary infiltrates into multiple cavitary lesions with air-fluid levels, followed by gradual regression of these lesions. Despite a prolonged clinical course and transient pleural effusions, the patient’s respiratory status steadily improved. He was transferred to a rehabilitation facility for functional recovery and was discharged without respiratory sequelae. Follow-up chest radiography two months later showed near-complete resolution of pulmonary abnormalities. This case highlights that even minimal kerosene aspiration can result in severe lung injury in adults, including cavitary changes. Despite the severity and prolonged course, favorable outcomes can be achieved with careful supportive management. Increased awareness of the risks associated with improper storage of hazardous substances is essential to prevent accidental ingestion, particularly in agricultural environments.

## Introduction

Kerosene aspiration can be a cause of chemical pneumonitis resulting from accidental ingestion, particularly in infants and children. However, cases involving adults are rare. Moreover, kerosene, a petroleum distillate, causes severe pulmonary injury when aspirated, characterized by inflammation and respiratory distress. Many reported cases in adults are associated with suicidal behavior, fire-eating practices, or occupational exposure [1,2].

Herein, we report a case of chemical pneumonitis following accidental ingestion of kerosene that had been stored in a plastic drink bottle during open-field burning. This case highlights the potential hazards of accidental kerosene aspiration in agricultural settings and the need for careful handling and storage of hazardous materials.

## Case presentation

A 73-year-old male farmer undergoing maintenance dialysis presented to the emergency department of our hospital with fatigue, dyspnea, and vomiting. The day before the presentation, the patient was engaged in farm work and had stored kerosene in a plastic drink bottle for open-field burning. He inadvertently ingested kerosene, having mistaken it for drinking water; however, he immediately recognized the error and spat it out. Despite remaining at rest, his symptoms did not improve.

**Figure 1:**
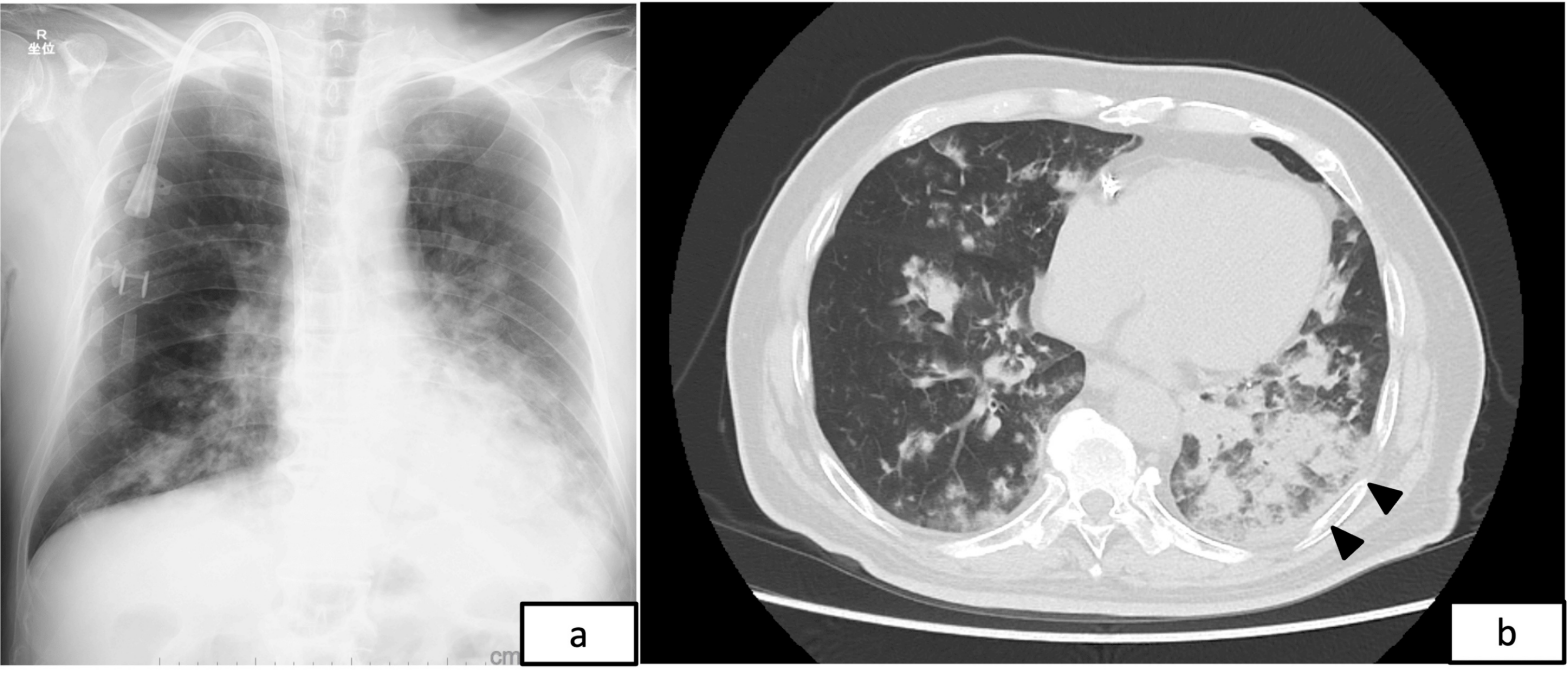
Chest radiograph (a) and computed tomography (b) on admission revealing bilateral infiltrative opacities, predominantly in the lower lung fields and bases. The hemodialysis catheter is visible on chest radiography.

The patient was tachypneic, with a respiratory rate of approximately 30 breaths per minute, and an SpO₂ of 92% on room air. Chest radiography and computed tomography (CT) revealed extensive pulmonary infiltrates predominantly in the lower lung fields (Figure 1). The patient was admitted to the hospital for the treatment of chemical pneumonitis. Upon admission, the patient was subsequently transferred to the intensive care unit for comprehensive management; however, mechanical ventilation was not required. Although the diagnosis was evident from onset, intravenous antibiotic therapy was initiated due to the potential for secondary bacterial infection. As there is no established specific treatment for kerosene aspiration, the patient was otherwise managed with supportive care and close observation.

**Figure 2:**
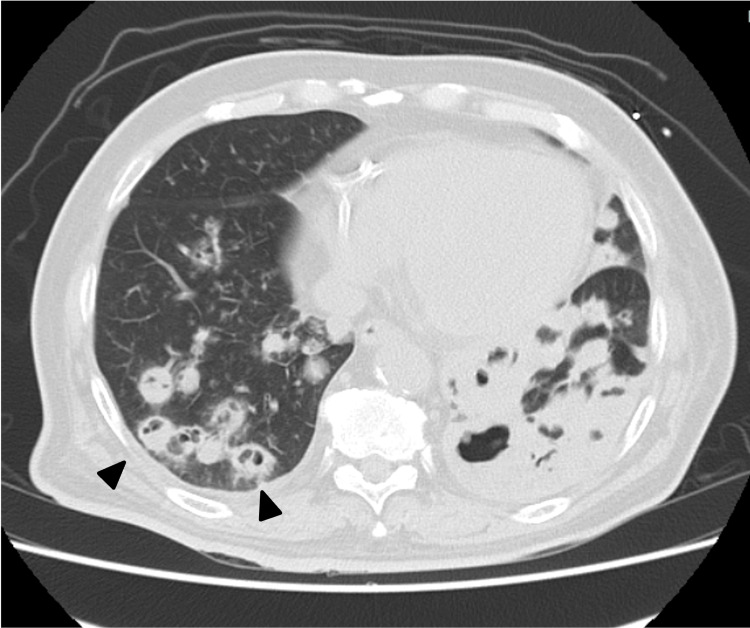
Chest computed tomography on Day X+7 displaying the development of multiple cavitary lesions in the previously infiltrated areas.

**Figure 3:**
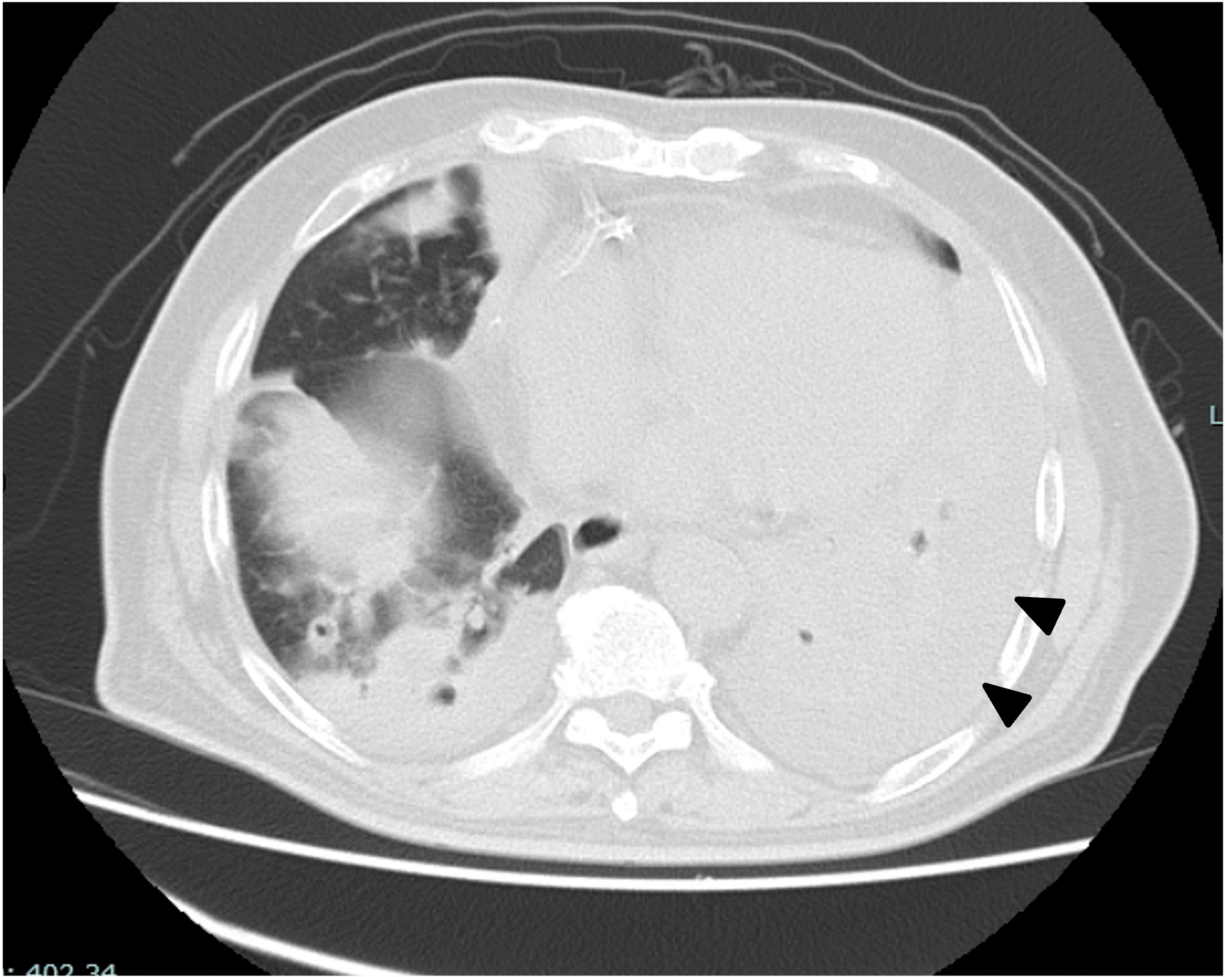
Chest computed tomography scan on Day X+21 demonstrating a reduction in the size of the cavitary lesions and pleural effusion occupying the thoracic cavity. Additional infiltrative opacities are observed, suggesting concomitant pneumonia.

On Day X+2, owing to persistent nausea, an upper gastrointestinal endoscopy was performed, which revealed only mild erythema and led to a diagnosis of corrosive gastritis. A chest CT performed on Day X+7 demonstrated partial resolution of certain infiltrates; however, multiple opacities exhibited cavitation with fluid accumulation within the cavities (Figure 2). On Day X+12, peripheral venous access became difficult, prompting a switch from intravenous to oral antibiotic therapy despite no definitive signs of infection. On Day X+21, worsening oxygenation prompted a repeat chest CT scan. The progression of some lesions raised suspicion of superimposed bacterial or fungal infections (Figure 3). The previously identified areas of lung collapse had reduced in extent, and bilateral pleural effusions were noted. Although the patient had a persistent low-grade fever of around 37℃, his overall condition had improved.

**Figure 4:**
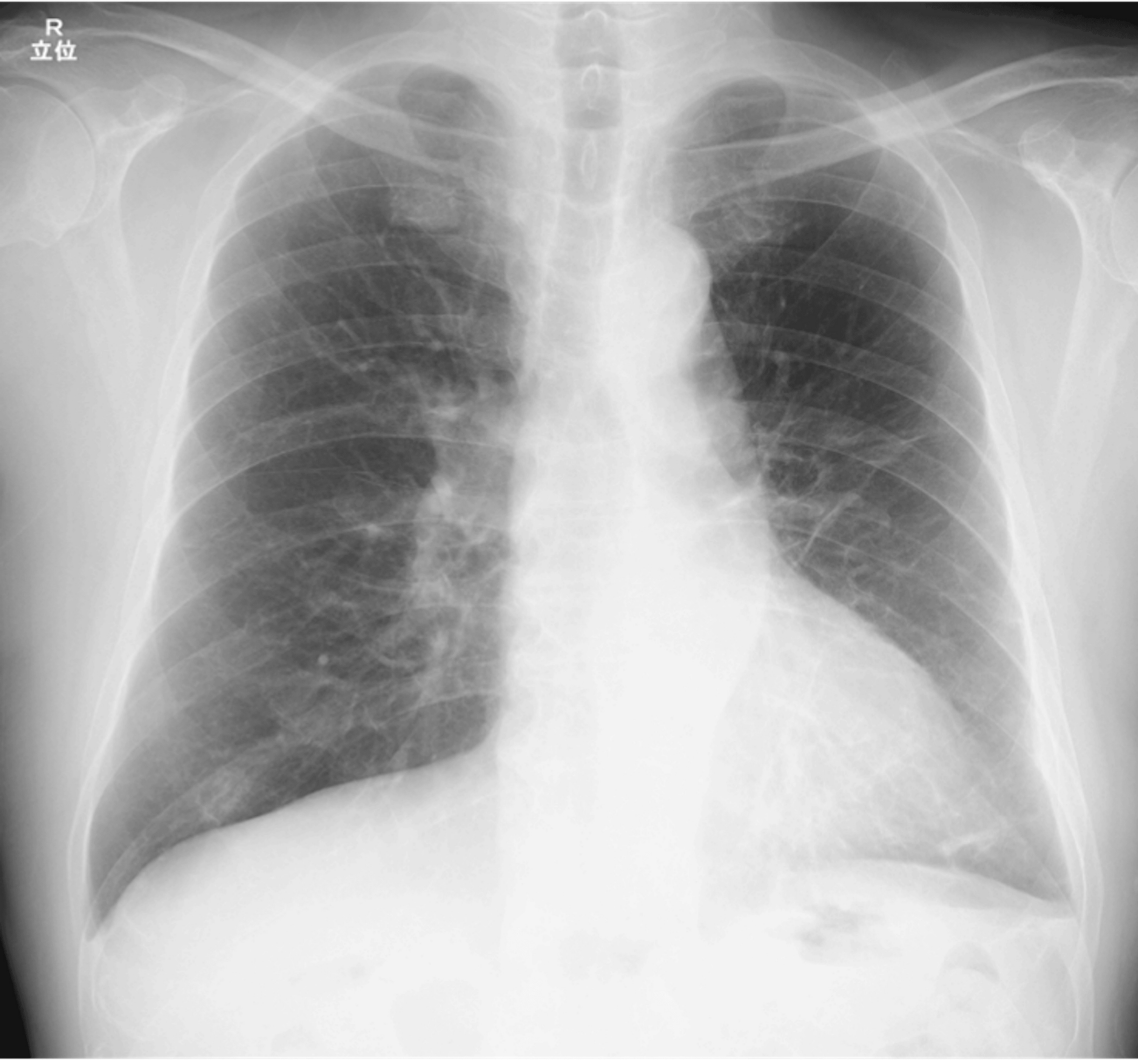
Chest radiograph on Day X+65 revealing near-complete resolution of the abnormal shadows.

Owing to functional decline following prolonged hospitalization, the patient was transferred to another facility for continued rehabilitation on Day X+43. At the two-month outpatient follow-up, chest radiography demonstrated near-complete resolution of previous abnormalities (Figure 4), and the patient’s symptoms had also resolved.

## Discussion

The lethal oral dose of kerosene is difficult to estimate; however, the systemic toxicity of kerosene is generally considered low, provided it remains within the gastrointestinal tract. Most fatalities are believed to result from chemical pneumonitis due to aspiration [3]; no reports of deaths attributable to other mechanisms were identified. Nevertheless, even small amounts can cause severe chemical pneumonitis, making the prediction of patient prognosis challenging based solely on the volume of exposure. When petroleum products such as kerosene are ingested, mucosal irritation can immediately induce coughing, nausea, and vomiting, and chemical pneumonitis is frequently observed [4]. Previous animal experiments demonstrated that gastric infusion of kerosene after esophageal ligation does not lead to increased pulmonary kerosene levels [5]. Thus, the potential for gastric contents to enter the airways must always be considered. Consequently, methods that induce vomiting are contraindicated in cases of kerosene ingestion; however, in the present case, spontaneous vomiting occurred, resulting in the development of severe chemical pneumonitis.

The unintentional oral ingestion of kerosene is extremely rare. Most reports in Japan involve children, and although cases of ingestion associated with suicide attempts have been described [6], we could not identify any reports of accidental ingestion comparable to the present case. Therefore, reports on chemical pneumonitis caused by kerosene aspiration in adults remain limited. Numerous reports have described chemical pneumonitis in fire eaters and street performers who unintentionally aspirate kerosene. A total of 123 cases were reviewed, of which 115 involved petroleum distillates [1], many of which were presumed to include kerosene. Among the 84 patients who ingested kerosene orally, respiratory symptoms (cough (42.0%), chest pain (40.7%), and dyspnea (17.3%) were more frequent than gastrointestinal symptoms such as nausea (35.8%). Although this represents a relatively large case series, caution is warranted in interpreting these findings, as fire eaters are chronically exposed in an occupational setting and are presumed to inhale vaporized rather than liquid components. These circumstances differ from those in the present case.

The present case involved a severe lung injury caused by kerosene aspiration. Bilateral cavitary lesions were observed, rarely described in previous studies. Rashed et al. reported a similar radiographic pattern in a three-year-old child with petroleum aspiration [2]. Diffuse infiltrates, predominantly in the lower lung fields, were followed by rounded lesions. These lesions progressed to cavitation with air-fluid levels by the second week, ultimately forming empty cavities by one month, presumably due to the discharge of their contents. This sequence, from infiltration to rounded lesions to cavitation, is highly similar to the clinical course observed in the present case. Cystic lesions are believed to originate from alveolar dilation secondary to necrosis of alveolar and bronchial structures, with disruption of the check-valve mechanism caused by the destruction of elastic tissue and necrotic epithelial cells, leading to air trapping [7,8]. However, previous reports on kerosene aspiration have not always described similar findings, suggesting that pulmonary pathology varies depending on the severity of exposure.

Rashed et al. reported four similar cases presenting with pulmonary volume loss and suggested, based on previous reports, that lesions are likely to improve within six to eight months [2]. In the present case, the cavity gradually decreased in size, resulting in expansion of the intrathoracic space, which was subsequently replaced by pleural effusion. However, chest radiography performed two months after discharge revealed near-complete normalization. Conversely, no pleural effusion was observed in the cases reported by Rashed et al. Instead, the lung parenchyma expanded to occupy the intracavitary space, and the cavities were nearly completely resolved by the two-month follow-up. This difference may be attributed to the fact that all cases reported by Rashed et al. involved pediatric patients in the growth phase, whereas the present patient was older, potentially contributing to differences in the clinical course. Nevertheless, the clinical course in the present case suggests that complete recovery without residual sequelae can be expected even in older patients.

Kerosene is classified as a volatile oil; however, its volatility is lower than that of gasoline, suggesting that the aspirated liquid components may cause persistent pulmonary injury. Vaporized kerosene is heavier than air and may remain in the lungs after evaporation. However, the extent to which vapor contributes to lung injury remains unclear. Thus, the potential benefit of washout using high-flow nasal cannula therapy for prognosis remains uncertain. Although some reports indicate that steroid therapy does not improve outcomes [9], corticosteroids are occasionally administered in clinical practice, particularly for the management of secondary acute respiratory distress syndrome [10].

In this case, inpatient management focused primarily on systemic supportive care and prevention and treatment of secondary infections. Although the patient experienced recurrent infections related to underlying alveolar destruction and was initially considered to have a poor prognosis, careful supportive management allowed these radiological abnormalities to be potentially reversible. Pulmonary infiltrates and cavitary changes gradually improved, and the patient ultimately achieved near-complete radiological recovery without long-term respiratory sequelae. Although reports of adult cases are limited, this case highlights that cavitary changes following chemical pneumonitis can be reversible with appropriate acute-phase management, providing an important clinical takeaway for practicing physicians.

## Conclusions

Kerosene aspiration can present as severe pneumonia with bilateral pulmonary infiltrates; however, no standardized treatment strategy has been established, and management is generally supportive. Although the prognosis has been reported to be favorable, most existing reports involve pediatric patients, and data on adult cases remain limited. Our case demonstrates that unintentional kerosene aspiration in adults may also have a favorable outcome with supportive care alone and without sequelae.
